# Online peer editing: effects of comments and edits on academic writing skills

**DOI:** 10.1016/j.heliyon.2022.e09822

**Published:** 2022-06-28

**Authors:** Han Zhang, Galina Shulgina, Mik Fanguy, Jamie Costley

**Affiliations:** a National Research University Higher School of Economics, Institute of Education, Myasnitskaya Ulitsa, 20, Moscow, 101000, Russia; b EFL Department, Korea Advanced Institute of Science and Technology (KAIST), 291 Daehak-ro, Guseong-dong, Yuseong-gu, Daejeon, South Korea

**Keywords:** Academic writing, Comments, Edits, Online collaborative learning, Peer feedback

## Abstract

Although the effects of online peer editing have been studied from a number of perspectives, it remains unclear how giving and receiving comments and edits affect student academic writing performance. The current study examined the influence of these aspects of peer editing on student academic writing performance in higher education during online peer editing. Participants were 76 students engaged in peer editing of one another's work in a graduate scientific writing course at a Korean university. The relationships between the giving and receiving of comments and edits, and student performance on their writing tasks were analyzed. Results showed that there is a positive correlation between the number of comments received and the student's writing score, whereas receiving edits had the opposite effect and was associated with lower student performance. Furthermore, no relationship was found between giving comments or edits and writing performance. These results add to the field's understanding of how specific elements of peer editing can impact students' performance.

## 1 Introduction

Due to the COVID-19 pandemic, blended and online learning have been used to provide students with diverse collaborative learning opportunities (Al-Samarraie and Saeed, 2018; Zhu and Liu, 2020). Existing research recognizes that peer feedback is a valuable tool for improving student academic performance (Al-Rahmi et al., 2015), thus interest has also grown in learner-to-learner interaction and how peer editing, as one type of collaboration, plays a role in interaction and student performance (Zhou, 2017). Peer editing is defined as a collaborative learning process during which peers interact, review, critique, and edit each other's work (Ebadi and Rahimi, 2017). It has been shown to be more effective than feedback from a teacher in some contexts (Cho and MacArthur, 2011; Ciftci and Kocoglu, 2012; Nicol et al., 2014). In the context of academic writing, both providing and receiving feedback may help students improve their writing skills as this kind of peer interaction allows students to gain knowledge from different perspectives through social sharing (Huisman et al., 2018). Furthermore, peer editing may lead both the giver and receiver of feedback to absorb information, and then decide how to judge the received messages through self-reflection (Casey and Goodyear, 2015). Therefore, students may take some measures to narrow the gap and reach their potential after the feedback interpretation (Carless and Boud, 2018; Wang et al., 2015; Zhu and Carless, 2018).

In online peer editing, providing comments or edits are the two most prevalent methods of providing feedback (Magnifico et al., 2015). Comments refer to offering opinions and leaving suggestions, usually using the embed comments function in Microsoft word or another type of word processing software. On the other hand, providing edits means making direct changes to student original text, which generally shows up as a different color than what the original author wrote in (Perron and Sellers, 2011). These two methods provide students an opportunity to discuss ideas and questions, review, criticize, and edit each other's work by adding suggestions and responding to them (Lin and Reigeluth, 2016; Zhu and Carless, 2018), which activate key cognitive processes. Existing evidence supports the claim that peer feedback may improve students' academic writing performance (Ebadi and Rahimi, 2017; Huisman et al., 2018, 2019; Zhang et al., 2021). However, there is a lack of clarity regarding how giving and receiving comments and edits will affect students' writing performance separately, especially in an online collaborative context. This study intends to explore the impact of comments and edits on students' writing performance from both giving and receiving perspectives.

## 2 Literature review

### 2.1 Two methods of online peer editing

As an in-class collaborative activity, giving and receiving peer feedback through online peer editing has been shown to greatly benefit student writing (Casey and Goodyear, 2015; Ebadi and Rahimi, 2017; Huisman et al., 2018, 2019). The enhancement that peer editing brings to writers seems to be beyond an improvement in the quality of a particular piece of writing. Engaging in peer editing helps students develop greater self-assessment skills when compared with editing alone (Nulty, 2011). It allows students to learn to critically review and revise their writing from the audience's perspective, thereby developing their independent thinking skills and self-directed learning (Ebadi and Rahimi, 2017). Through communication and interaction with their peers, students become more actively engaged in their own writing (Nicol and Macfarlane-Dick, 2006).

Online peer editing allows students to offer comments or edits to their peers. Specifically, comments refer to the leaving suggestions to identify the strengths and weaknesses of their peers, while edits are the act of inserting and/or deleting text written by other students (Liu and Edwards, 2018). Once the editor has made the edits or left comments, the author of the text may respond to the comment, mark it as resolved, or delete it. In terms of edits of others, students can delete words and phrases directly, correct others’ spelling mistakes and add sentences or paragraphs. Compared to comments, edits provide direct changes without supporting arguments, which may, in some cases, prevent the author of the text from understanding the reasons for the proposed solution and, in turn, cause the author to decline it (Liu and Edwards, 2018). Furthermore, the writer can often accept the changes without checking or understanding why the changes were made. Understanding the problem is an important predictor of effective feedback implementation (Nelson and Schunn, 2009).

### 2.2 The influence of receiving comments on learning

Many studies have shown improvements in performance after students received comments during the learning process (Huisman et al., 2018, 2019; Zhang et al., 2021). A possible explanation is that receiving comments from others is helpful to student learning because during this process, receivers are encouraged to participate in the evaluation and reflection of their peers' comments (Shvidko, 2015). Students may improve themselves as their peer has identified the pros and cons of their essays, and then the author may think about whether they agree with those opinions and find solutions to solve the problems noted by the reviewer (Nicol et al., 2014).

However, it is not always the case that receiving comments will lead to improvements in writing or learning performance. For instance, some research has pointed out that receiving summaries, explanations, or ideas in comments is more helpful to student writing than some direct praise or criticism (Wu and Schunn, 2020). Sometimes, students feel less motivated when they receive comments with no supporting evidence, which reduces the potential benefits of this type of peer activity (Zhang and Hyland, 2018). Furthermore, comments may be ineffective if students do not consider, organize or fully implement them during the reflecting process (Nicol and Macfarlane-Dick, 2006). Holmes and Papageorgiou (2009) suggested that if the comments students receive are of low quality or the allocation is not appropriate, they will not help students' writing. Thus, there is a lack of clarity on how different received comments affect students’ writing.

### 2.3 The influence of giving comments on learning

How giving comments might affect academic writing is another area of required investigation that is within the scope of peer feedback. There is some evidence that giving feedback may be even more important than receiving it (Ion et al., 2019; Rouhi and Azizian, 2013). Students may develop their critical thinking abilities and metacognitive strategies through providing comments, and in some cases, their ability to problem-solve can be increased to a greater degree than those who receive feedback during the process of writing (Cho and Cho, 2011; Frank et al., 2018). Through this process, students may explore ideas collaboratively and focus on the connections between ideas while seeking to improve their writing (Neumann and McDonough, 2015).

During this process, students may produce, present, and develop their knowledge of a certain topic and share that knowledge with another learner whose work they are giving feedback on (Tai et al., 2018). Furthermore, many studies have stated that generating explanations is an effective method to improve one's own writing (Dunlosky et al., 2013; Nicol et al., 2014; Tempelaar et al., 2015). For instance, Cho and MacArthur (2010) pointed out that when students try to give an explanation by themselves, it is much more useful than receiving them from an expert. Those findings revealed that judging from a given text can enhance students' writing performance (Henderson and Phillips, 2015).

However, effective feedback necessitates deliberate coordination between the provider and recipient in a peer feedback-friendly atmosphere (Ray and Singh, 2018). In general, comments as a type of peer feedback should be provided only when the learner welcomes it (Jug et al., 2019). In some cases, certain comments can incite social conflicts in groups so that some comments givers may try to offer praises or other kind suggestions to avoid conflicts within groups, which may not lead to the best learning outcomes (Fong et al., 2018).

### 2.4 Receiving edits and student performance

Research has revealed that students can gain insights into their collaborators’ views on their work by reflecting on edits that others make of their work (Mabbott and Bull, 2006). According to the quality of the edits and where they are directed, students can begin to understand the quality of their own work (Hattie and Clarke, 2018). Furthermore, edits can range from simple superficial corrections, such as grammar or spelling errors, or more elaborate and deeper changes directed at the conceptual and knowledge level; after receiving and judging these edits, students may be more sensitive to avoid the same mistakes in their writing (Petrović et al., 2017).

On the one hand, highlighting spelling or grammar mistakes helps to improve the quality of writing. On the other hand, getting only grammatical edits as feedback may make students question their peers' abilities and cause feelings of disappointment (Birnholtz and Ibara, 2012; Liu and Edwards, 2018). In general, students believe that collaborative writing and peer editing lead to better quality work. However, while they may perceive their edits and suggestions as a source for improvement for others' texts, sometimes received edits may be seen as an intervention which makes their texts worse (Blau and Caspi, 2009). Also, edits often only highlight mistakes, which makes students perceive them as direct criticism, which may be harmful for subsequent improvement of students' writing (Tseng and Tsai, 2007), lower their sense of psychological ownership (Blau and Caspi, 2009), or lead to conflict (Birnholtz and Ibara, 2012). Interestingly, a high number of modifications may hurt students' feelings, while a small number of edits makes them feel uninvolved by their classmates. A lack of edits may be interpreted by students as an indication of disengagement or disinterest, which could negatively impact students’ writing (Mabbott and Bull, 2006).

### 2.5 Giving edits and student performance

Many studies have confirmed the effectiveness of online editing among peers, such as edits of others, for improving their active learning, as discussed above regarding comments (Wang, 2015). For instance, reading others' writing and correcting their errors can motivate them to seek information about what they are reading about or double-check their own understanding of concepts they are reviewing (Wang, 2015; Yen et al., 2015).

However, several circumstances, such as a lack of expertise with peer editing in general and online learning in particular, may have a detrimental impact on the usefulness of edits in improving the student writing performance. According to Ishtaiwa and Aburezeq (2015), opaque criteria for peer editing and the lack of information about the expected level of contribution may lead to minimal and/or overly formal student participation. Some students may also have difficulties with using some functions of the learning environment as a new instrument for learning, which can limit their participation (Ishtaiwa and Aburezeq, 2015). Moreover, students often may try to avoid editing their peers’ work because of the risk of upsetting the author (Birnholtz and Ibara, 2012; Coyle, 2007).

### 2.6 Present study

The present study seeks to explore the influence of giving and receiving peer feedback in the form of comments and edits on student learning. This study looks at the total amounts of comments and edits and does not investigate the quality or function of comments or edits. The reasons for this is that the first step in this type of research agenda is to look at the impact of the quantity of peer feedback elements on student writing performance. This gives a broad overview of how comments and/or edits impact student performance. This is a necessary first step in the understanding of how peer-to-peer feedback behaviors have on author and editor performance. Furthermore, when dealing with a high volume and number of students, as well as the use of technology for statistics and analysis, the amount of peer feedback is easier to obtain than the quality. This means that the outputs of comments and edits are readily available in the form of learning analytic visualizations more so than measures of edit or comment quality and function. Furthermore, this type of information can be more potential ready use for instructors to better understand online peer editing and implement instructional design choices that may help students improve their writing skills.

To achieve this, the present study collects data on comments and edits from peer editing sessions from 76 students over 5 cases of peer editing. Since most extant research explored the role of peer editing in broad ways such as surveys or interviews, the field has not yet dug deeply into how the volume of different peer editing methods influence students' learning performance. This study looks at peer editing by measuring comments and edits in students’ written documents directly in a collaborative learning context. To measure the performance of students, the overall individual writing scores are representative of the students' learning outcomes and performance during the course. The existing literature discussed above suggests that, on balance, giving and receiving comments and/or edits will lead to better learning performance and based on this, the present study has four main hypotheses:

H1 Students who receive more comments will perform better in their writing.

H2 Students who give more comments will perform better in their writing.

H3 Students who receive more edits will perform better in their writing.

H4 Students who give more edits will perform better in their writing.

## 3 Methodology

### 3.1 Participants and learning context

There were 76 students engaged in peer editing of each other's work in 4 sections of a graduate scientific writing course at a Korean university. Each of the 4 course sections had between 16 to 22 students. Among the subjects, 49 subjects were master's students, and 27 were in a doctoral program. There were 22 females and 54 males. The average age of the students was 25.7 (SD = 3.6), with a minimum age of 21 and a maximum age of 39 among the participants in the present study. The purpose of the scientific writing course was to teach students to write a journal manuscript on their graduate research findings (Zhang et al., 2021). The course was given in an online format, and pre-recorded video lectures were provided on the course learning management system for students to view at their convenience. The course consists of 10 instructional weeks that respectively include 4 to 8 lecture videos, totaling 56 lecture videos for the course. The average length of a course video is nearly 12 min and covers topics related to scientific writing for graduate STEM students.

The ten instructional weeks of the course were grouped into two-week units designed to provide instruction related to the five major sections of a journal manuscript: 1) Introduction, 2) Methodology, 3) Results, 4) Discussion & Conclusion, and 5) Abstract. In the first week of a given unit, students would watch a set of videos specific to the journal section of interest for that unit. Videos in this first week explained the purpose, function, characteristics, and conventions of the given section of a journal manuscript. After viewing this initial set of videos, students would attend a live session of the course with the course instructor using Zoom teleconferencing software. After leading a short discussion on the topics of the course videos and answering any student questions, the instructor would put students into small groups for collaborative learning activities to reinforce their learning regarding the concepts covered in the lecture videos.

The second week of the unit consisted of another set of lecture videos often providing instruction on writing style, language, and grammar related to the same manuscript section focused on in the first week of the unit. Prior to the Zoom meeting of the second week of the unit, students were instructed to compose a first draft of a journal article section of focus in the unit and bring it to the meeting. While no special instructions were given to students in terms of word count, students were advised to consult journal style guides and published papers in their fields of study in deciding on the length and format of their written assignments. At the Zoom meeting, the instructor led a short discussion and answered questions and then provided instruction for the peer editing session. Then, students grouped themselves into dyads, and the instructor moved them into breakout rooms so that they could peer edit one another's writing. In the first week of the semester, students filled out a short questionnaire on their major, degree program, areas of research interest or expertise, and the title of their research project or paper. This information was shared with the class through a spreadsheet so that students could choose a peer editing partner with research interests that were as aligned as possible with their own. Ethical approval from a KAIST Institutional Review Board (IRB) named “The Effects of Collaborative Notetaking on Learning Outcomes in Online and Blended Learning Environments'' was received before conducting the questionnaire.

A Google Doc was created by the course instructor for each member of the dyad for each of five peer editing sessions, which corresponded to the Introduction, Methodology, Results, Discussion & Conclusion, and Abstract sections. Students were instructed to copy and paste their first draft of the journal manuscript section into the corresponding peer editing Google Doc and to share with and provide editing privileges to their dyad member. The peer editing Google Doc contained instructions for the students on how to peer edit their partner's work, and a video on how to peer edit an assignment was provided on the course learning management system. The instructions provided in the video and within each peer editing Google Doc required the students to track any changes to their partner's paper using “suggesting mode” rather than “editing mode”. This was done so that the original author of the work could easily see any changes that were made and would easily be able to accept or reject any changes according to preference. In addition, students were encouraged to make use of the embedded comment feature within the Google Docs platform, which allows a collaborator to highlight a given section of text and embed a comment that shows up in the right margin of the document. Replies to such comments are possible, so that the author and editor can engage in a comment thread if they desire. After providing such edits and comments, reviewers were asked to grade the quality of the draft using a specialized rubric provided by the course instructor adapted from Clabough and Clabough (2016). The rubric assessed five criteria: four criteria specific to the content and function of a given section of a journal article and one general criterion related to the clarity and readability of the writing, and allowed students to rate the quality level of each criteria as “poor,” “average,” or “excellent”, giving scores of 0, 1, and 2, respectively. Accordingly, these subscores were added up and amounted to a final score from 0 to 10.

At the end of this second live Zoom meeting for a given unit, students were instructed to consider their partner's feedback on the first draft of their assignment and to create a final draft for submission on the course learning management system for final grading by the course instructor. Students were given two days to complete the revision, and the course instructor provided comments, suggestions, edits, and a final grade out of 10 points using the same specialized rubric that was used for peer editing. This 10-point grade accounted for 10% of the student's final grade in the course. Completion and grading of the final draft marked the end of a given unit, and the following week would begin a new unit of the course until all sections of the paper were completed.

### 3.2 Research instruments

**Comments.** Students can develop critical thinking skills, improve the structure of their writing, and gain new insights and perspectives when provided with written comments from their classmates (Sung et al., 2016). In the present study, *comments* refer to written feedback students receive from a peer editing partner on their individual writing using embedded commenting features within the Google Docs platform. Such embedded comments appear as small frames in the margin of the document. Prior research has shown that embedded comments can be used to provide feedback and assessment at a variety of levels, from superficial, such as grammar and spelling, to highly complex, including deeper conceptualizations and connections of knowledge (Luo et al., 2016; Strijbos and Wichmann, 2018; Sung et al., 2016). Embedded comments can also be used by editors and coauthors to ask questions and engage in online discussions in the margins of the document. For the purposes of this study, the number of embedded comments and replies to comments within a given peer editing Google Doc serves as the *comments* variable.

**Edit of others.** When peer editing one another's writing, students change or delete the writing of the original author of a text. In prior research, an increase in such *edits of others* was shown to correlate positively with students' ability to write clearly and to support their claims with evidence (Yim et al., 2017). The *edits of others variable i*s the total number of characters inserted by a collaborator after the collaborator deleted text from the original author. This definition was originally provided by Wang et al. (2015) in their paper presenting DocuViz, an add-on for Google Chrome that enables collaborative data, including edits of others, to be mined and visualized from Google Docs. In the present research, DocuViz was used to mine this editing data from each of the peer editing Google Docs.

**Writing assignments.** The primary assignments for the scientific writing course examined in this study were the five major sections of a research manuscript: 1) Introduction, 2) Methodology, 3) Results, 4) Discussion & Conclusion, and 5) Abstract. Using a rubric adapted from Clabough and Clabough (2016), these writing assignments were assessed by the course instructor and given a grade from 0 to 10. These assignment grades were then tallied to give a total writing score out of 50 points, accounting for 50% of the total grade points for the course.

## 4 Results

Correlational analyses were conducted to examine the connection between the variables and then evaluated for significance to test the hypotheses. The first step of looking into the research questions was an overview of the main variables that were used as a part of this study. As can be seen in Table 1, the authors wrote on average 2588 words, with the longest piece of work being 6829 words, and the shortest being 410 words. The students performed well in regards to their writing score, with the average score being 41 out of a possible 50. Also, worth noting is the lowest writing score attained in the sample population (32) is considered a passing grade for the writing portion of this class. The comments received and comments given have the same mean score of 11.16 embedded comments as these variables are the inverse of each other. As with comments, the edits received and edits given number of key-strokes have the same mean, which in the case of edits was 3881.38 keystrokes.

**Table 1:** Descriptive statistics for the main variables used in the study.

	Min	Max	Mean	SD	Description
Author volume	410	6829	2587.68	1354.07	Words
Writing score	32	50	41.36	3.96	Rubric based score
Comments received	0	41	11.16	10.93	Embedded comments
Edits received	10	30818	3881.38	5539.64	Keystrokes
Comments given	0	41	11.16	11.16	Embedded comments
Edits given	0	30818	3881.38	5539.64	Keystrokes

To look more closely at the variables that could be analyzed as a part of this study, correlations between all main variables as well as author volume were calculated (Table 2). The results show that receiving comments had a statistically significant positive association with writing score (.232∗). In contrast, receiving edits had a negative statistically significant association with writing score (-.325∗∗). In regards to the giving of comments and the giving of edits, neither variable had a statistically significant relationship with writing score.

**Table 2:** Correlations between all variables.

		1	2	3	4	5	6
1	Author volume	1					
2	Writing score	.521∗∗	1				
3	Comments received	.466∗∗	.232∗	1			
4	Edits received	.018	-.325∗∗	-0.19	1		
5	Comments given	.162	.087	.485∗∗	-.117	1	
6	Edits given	.270∗	-.068	-.118	.327∗∗	-.181	1

∗∗Correlation is significant at the 0.01 level (2-tailed).

∗Correlation is significant at the 0.05 level (2-tailed).

Notable on this table are the positive associations between author volume and writing score (.521∗∗), comments received (.466∗∗) and edits given (.270∗). This shows that authors who produced more words had higher writing results. Furthermore, individuals who wrote more words encouraged their peers to comment on them more. Finally, people who wrote more appeared to be more likely to edit the work of others. Another finding that can be seen in this table is the positive relationship between giving comments and receiving comments (.485∗∗). Furthermore, there was also a positive relationship between receiving edits and giving edits (.327∗∗). These two results suggest that pairs tended to fall into a pattern of engaging in the same types of peer-editing - either commenting, or editing.

## 5 Discussion

Although previous research has investigated online peer editing, it remains unclear how the giving and receiving of comments and edits affect student writing performance. The current study explores the influence of these aspects on student academic writing performance in higher education by using Google Docs. The results show that receiving comments is positively associated with student writing performance. However, receiving edits has a negative association with student writing. In terms of giving comments and giving edits, neither technique has a statistically significant association with writing performance.

The findings indicate that students who receive more comments will write better papers, which coincides with evidence suggesting that students may improve their writing performance after receiving comments from their peers in the learning process (Ebadi and Rahimi, 2017; Huisman et al., 2018, 2019; Shvidko, 2015; Zhang et al., 2021). Students may improve their work based on the feedback they received, which identifies their writing's strengths and faults (Casey and Goodyear, 2015). Students may identify answers to difficulties raised by the reviewer because of self-reflection, and their writing may improve as a result (Nicol et al., 2014). This finding may be also due to the positive perceptions and attitudes of the learners because looking at others' comments through Google Docs provides learners with enough time and space to think, judge, and choose to accept or reject these suggestions, and eventually enhance their writing skills (Ebadi and Rahimi, 2017). All students are masters and PhD in present study; thus, student writing may be in high quality and students may be more likely to receive higher quality comments from their high-performing peers. On the contrary, some research revealed that not all kinds of comments are effective (Jug et al., 2019; Ray and Singh, 2018). One possible interpretation of this statement is that some comments are not well-structured or well-considered, so that students refuse to accept them. However, this research found that comments as suggestions and effective learning resources are useful to enhance student academic writing. As a result, comments should be expressed directly and clearly, and they should be comprehended independently of the giver; otherwise, receivers may not be able to grasp them, becoming confused and, in some cases, refusing to provide feedback (Hattie and Clarke, 2018; Holmes and Papageorgiou, 2009).

Another finding of the present study is that giving comments during online peer editing has no association with students' writing performance. This contrasts with previous literature where giving comments to their peers can promote students' critical thinking and metacognitive strategies, thereby enabling improvement of their writing skills (Cho and Cho, 2011; Frank et al., 2018; Dunlosky et al., 2013; Nicol et al., 2014). The finding in the present study may be related to the concerns of some feedback givers. For instance, students may try to avoid social conflicts within groups by only giving praise or soft advice to others, which may lead them to not engage personally with others’ work (Fong et al., 2018; Robertson, 2011). Therefore, perhaps instructions are needed to guide students on how to deliver comments at deeper levels to improve writing before conducting online peer editing so that the givers of comments can also benefit from peer editing.

The most surprising result of the present study also shows that receiving edits negatively correlates with student writing performance, suggesting that such behaviors may stop students from improving their writing skills. This finding seems to contradict the work of Mabbott and Bull (2006), who claimed that students will be more sensitive to their mistakes after reading and judging the edits from their peers, which should lead to better performance. However, the negative correlation between receiving edits and student writing is in line with Liu and Edwards (2018), who illustrated that students may be upset or question the ability of their peers when they only received edits related to grammar or spelling errors. In turn, the effects of peer editing may rely on the quality of the reviewed writings (Cho and Cho, 2011). When the editors look through a paper of poor quality, too many grammar and spelling errors make it difficult to give in-depth edits, thereby limiting the effects of edits on their peer's writing. For low-quality writing, it is possible that their peers are confused about the writing itself so that they are not able to offer edits. Interestingly, the average amount of edits received by students was 3881.38 characters. Receiving such large volumes of edits during collaborative learning may increase the workload of students' reflection, and students may only choose to accept all suggested edits without considering their accuracy, which may lead to a worse outcome in writing performance.

It is suggested by the results of the current study that giving more edits in groups does not drive better writing performance. It refutes the claim that reviewing others' sentences and pointing out their mistakes is a useful method to look for information and double check their own understanding of concepts mentioned by their peers (Wang et al. 2015; Yen et al., 2015). This result likely has several causes. The first is that students may lack experience in using online collaborative technologies and giving edits online through Google Docs, which may hinder them from participating in online peer editing and gaining benefits from it (Ishtaiwa and Aburezeq, 2015). Ludemann and McMakin (2014) found negative relationships between the first experience as a peer editor and assignment grades; however, for the second and subsequent sessions, this correlation did not hold. In the present study, five peer editing sessions were conducted. According to Jeffery et al. (2016), the accuracy of peer editing is greatly impacted by the number of reviewers, and they suggested that there should be at least three reviewers for one academic paper. Therefore, interaction between two people may influence the results, especially if the feedback givers have little experience in peer editing. The second possible explanation for the negative association found in the present study is that students try to edit others’ sentences kindly and superficially to avoid upsetting the author (Birnholtz and Ibara, 2012; Coyle, 2007). This type of editing may distract the author without any benefit as the edits are superficial and not helpful in increasing writing performance.

There are also some other interesting findings in the present study. For instance, positive correlations were found between author volume and their writing scores, comments received, and edits given. It is possible that when students have a well-rounded understanding of the topic, they may hold a positive attitude and prefer to express more in their writing. In turn, they will get higher scores than those who did not put a lot of effort into writing assignments. In addition, when the author volume becomes higher, there are more materials that can be provided to their peers for feedback, so that students may offer more comments and edits to their peers (Nicol et al., 2014).

There is also a positive relationship between comments received and comments given, and between edits received and edits given. When considering the influence of peer review, one should remember that every student is both a reviewer and a writer (Cho and Cho, 2011). Specifically, students act as reviewers to comment or edit on others' drafts, and as authors, they receive comments or edits from other reviewers’ perspectives (Cho and Cho, 2011). Thus, the effect of receiving comments/edits and the role of giving comments/edits need to be considered together.

## 6 Conclusion

The aim of this research was to explore how giving and receiving comments and edits influence students' writing performance. Since previous studies used broad methods to investigate students' perspectives on peer editing, this paper fills a gap in the research on online peer feedback by categorizing feedback as comments or edits and separately examining them in documents. One of the contributions of the present study is that it reveals receiving comments during online peer editing is a useful method to improve student writing performance, which provides empirical evidence that judging and reflecting on received comments enables students to enhance their writing skills. Interestingly, another finding from this research showed that there is a negative correlation between receiving edits and students' writing performance, which may be due to the large volume of edits and the low level of students’ writing abilities. However, there is no statistically significant correlation between giving comments and/or edits and student writing performance. These findings suggest two important recommendations for instructors facilitating online peer editing sessions: 1) encourage students to participate in self-reflection after receiving comments actively and 2) provide some instructions before peer editing on how to give deeper levels of comments and edits during online peer editing. For example, before online collaboration, instructors can show students the example of good peer feedback and point out what types of feedback can enhance their writing.

Since online learning has become increasingly popular, the present research is particularly relevant as it suggests new avenues for improving students' online writing performance in online settings. However, there are some limitations to this research. For instance, in this study, students could choose their own partners rather than having partners randomly assigned to conduct online peer editing. In this case, students may prefer to give kind suggestions or less edits to protect their friends' feelings, which may have a negative influence on the outcome of this research. Thus, future research should assign students to different groups randomly to increase the validity of research. Another limitation is that while this study accounts for edits and comments that were provided by peers within a Google Doc, it does not account for backchannel communication occurring outside the document, including discussions during Zoom video conferencing while the peers edited each other's’ work or subsequent communications proceeded via email or text messaging. While such backchannel communications would likely provide a rich source of data on students' collaborative processes, the collection of such information would be invasive to students' privacy and is not allowed by the institutional review board that granted permission for the present study. One other limitation is that the present study only took into account the number of comments and modifications, not their intention or quality. More extensive research in the future might take into account both the quantity and quality of comments and edits. More study could be done to develop a systematic method for accounting for these two factors. Although previous research has investigated the influence of peer editing on student academic writing, further exploration is needed on how giving and receiving peer feedback online affect students' writing. Since comments and edits, two popular modes of online editing, play an essential role in cooperative learning, more research is needed to explore the impact of peer editing in online contexts.

## Declarations

### Author contribution statement

Han Zhang: Conceived and designed the experiments; Wrote the paper.

Galina Shulgina: Contributed reagents, materials, analysis tools or data; Wrote the paper.

Mik Fanguy: Performed the experiments.

Jamie Costley: Analyzed and interpreted the data.

### Funding statement

This research did not receive any specific grant from funding agencies in the public, commercial, or not-for-profit sectors.

### Data availability statement

The authors do not have permission to share data.

### Declaration of interests statement

The authors declare no conflict of interest.

### Additional information

This article is an output of a research project implemented as part of the Basic Research Program at the National Research University Higher School of Economics (HSE University). Supplementary content related to this article has been published online at doi: https://doi.org/10.1016/j.heliyon.2022.e09822.

## Appendix A Supplementary data

Notetaking Survey Notetaking Survey
