# Supplementary material for: Online peer editing: effects of comments and edits on academic writing skills
Source: Heliyon. 2022 Jun 28;8(7):e09822. doi: 10.1016/j.heliyon.2022.e09822 (PMC9254337; doi:10.1016/j.heliyon.2022.e09822)
Supplement: Notetaking Survey [file mmc1.docx]

**Notetaking Survey**

This survey will help us to understand your experience with online learning at KAIST. You will never be identified in any way, and your decision to participate or not will have no effect on your grade in this class. The contents of your research writing will NOT be included in the data collected for this study. This is a survey about your experiences and preferences regarding peer editing. Your data will be anonymized and stored in a secure repository. The completion of this survey will help us to understand you better.

* Required

1. Do you allow the researchers to use this survey and data produced as part of this class for the purposes of research? *

Yes

No

1. What is your student ID? *
2. What section of Scientific Writing are you taking? *

Section B

Section D

Section G

Section J

Section M

Section Q

Section S

Section T

1. What is your nationality? *
2. Writing the first draft of my assignment was extremely difficult due to my lack of knowledge and/or experience. *
3. 2 3 4 5 6 7

Not at all true of me

Very true of me

1. Instruction provided through this course was helpful in writing my paper. *
2. 2 3 4 5 6 7

Not at all true of me

Very true of me

1. Video instruction I received was instrumental to my revisions leading to the second draft of my assignment. *

1 2 3 4 5 6 7

Not at all true of me

Very true of me

1. Peer feedback that I received was instrumental to my revisions leading to the final draft of my document. *

1 2 3 4 5 6 7

Not at all true of me

Very true of me

1. Instructor feedback was instrumental to my revisions of my paper before submitting my work for publication in a journal. *
   1. 2 3 4 5 6 7

Not at all true of me

Very true of me

Is there anything further you'd like to add or comment about regarding peer editing or feedback?

**Thank you for completing the survey**
